# Supplementary figures and images for: Sex differences in cachexia outcomes and branched-chain amino acid metabolism following chemotherapy in aged mice
Source: PLoS One. 2026 Jan 12;21(1):e0340647. doi: 10.1371/journal.pone.0340647 (PMC12795360; doi:10.1371/journal.pone.0340647)

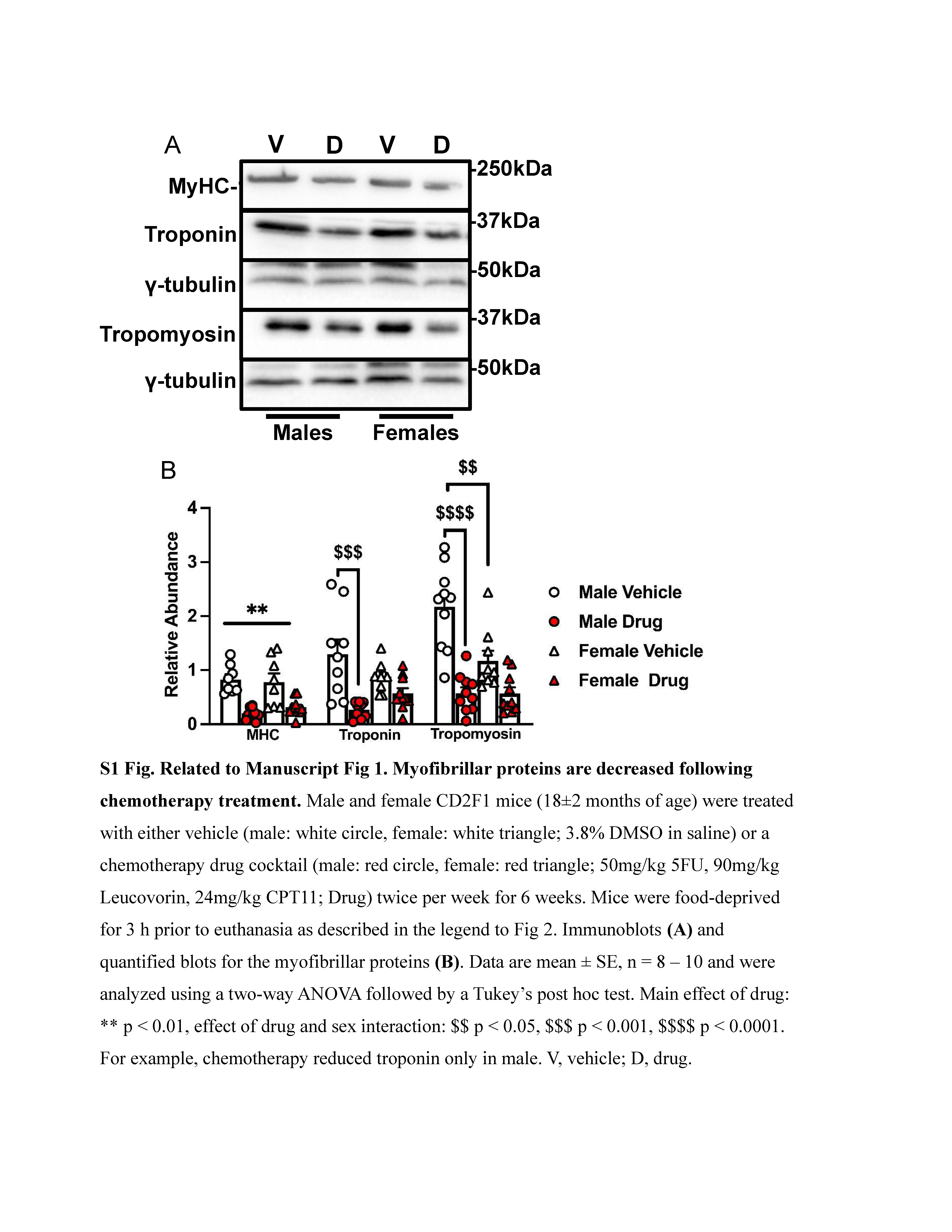

Supplement: S1 Fig — Myofibrillar protein levels are reduced following chemotherapy treatment. Male and female CD2F1 mice (18 ± 2 months old) were treated with either vehicle (Vehicle, V; males: white circle, females: white triangle; 3.8% DMSO in saline) or a chemotherapy drug cocktail (Drug, D; males: red circle, females: red triangle; 50 mg/kg 5FU, 90 mg/kg Leucovorin, 24 mg/kg CPT11) twice per week for 6 weeks. Immunoblots (A) and quantified blots for myofibrillar protein levels in the gastrocnemius muscle (B) are shown. Data are mean ± SE, n = 8–10 and were analyzed using a two-way ANOVA followed by a Tukey’s post hoc test. Main effect of chemotherapy: ** p < 0.01, interaction effects of chemotherapy and sex: $$ p < 0.05, $$$ p < 0.001, $$$$ p < 0.0001. For example, chemotherapy reduced troponin only in males. (TIF) [file pone.0340647.s001.tif]

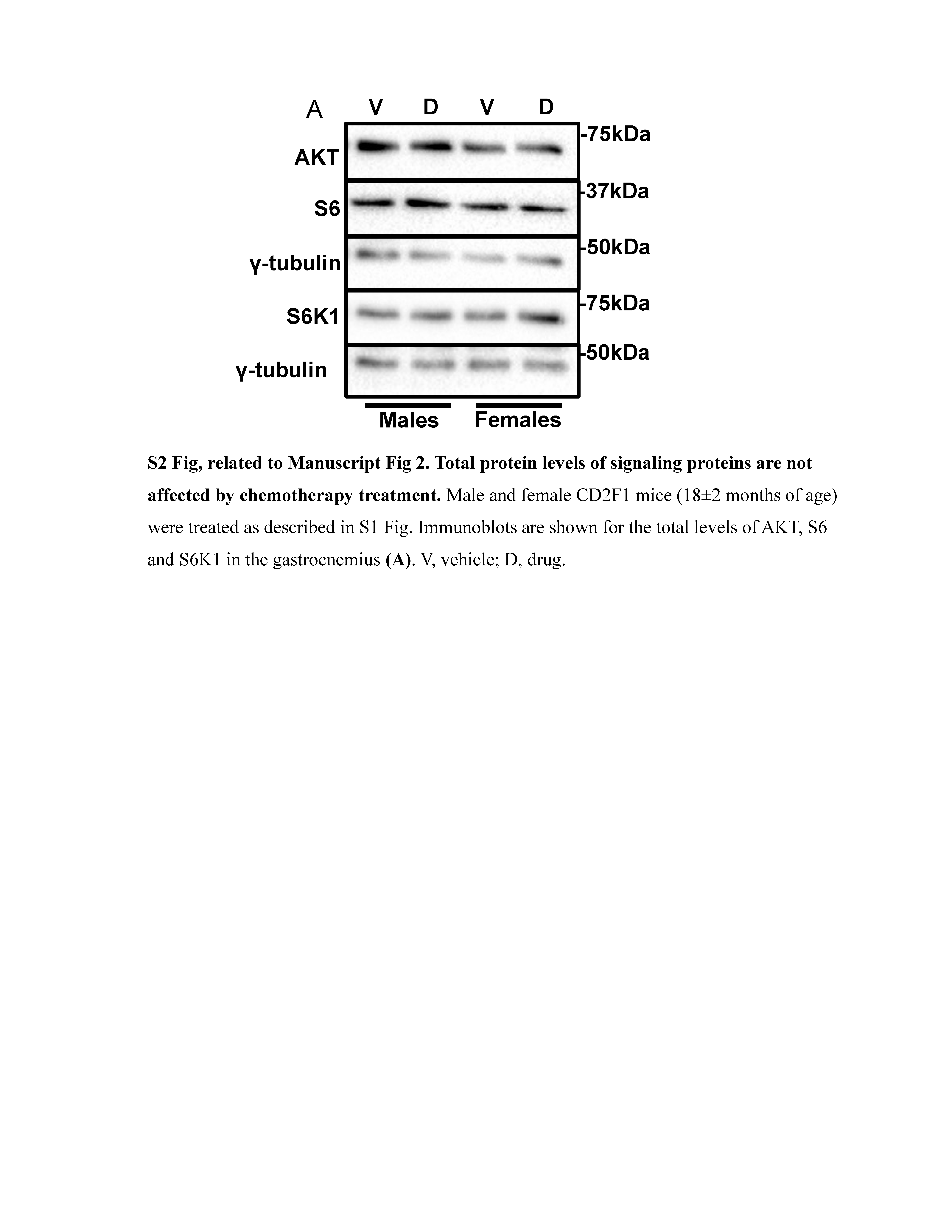

Supplement: S2 Fig — Total protein levels of signaling proteins are not affected by chemotherapy treatment. Male and female CD2F1 mice (18 ± 2 months old) were treated as described in S1 Fig. Immunoblots are shown for the total levels of AKT, S6 and S6K1 in the gastrocnemius muscle (A). (TIF) [file pone.0340647.s002.tif]

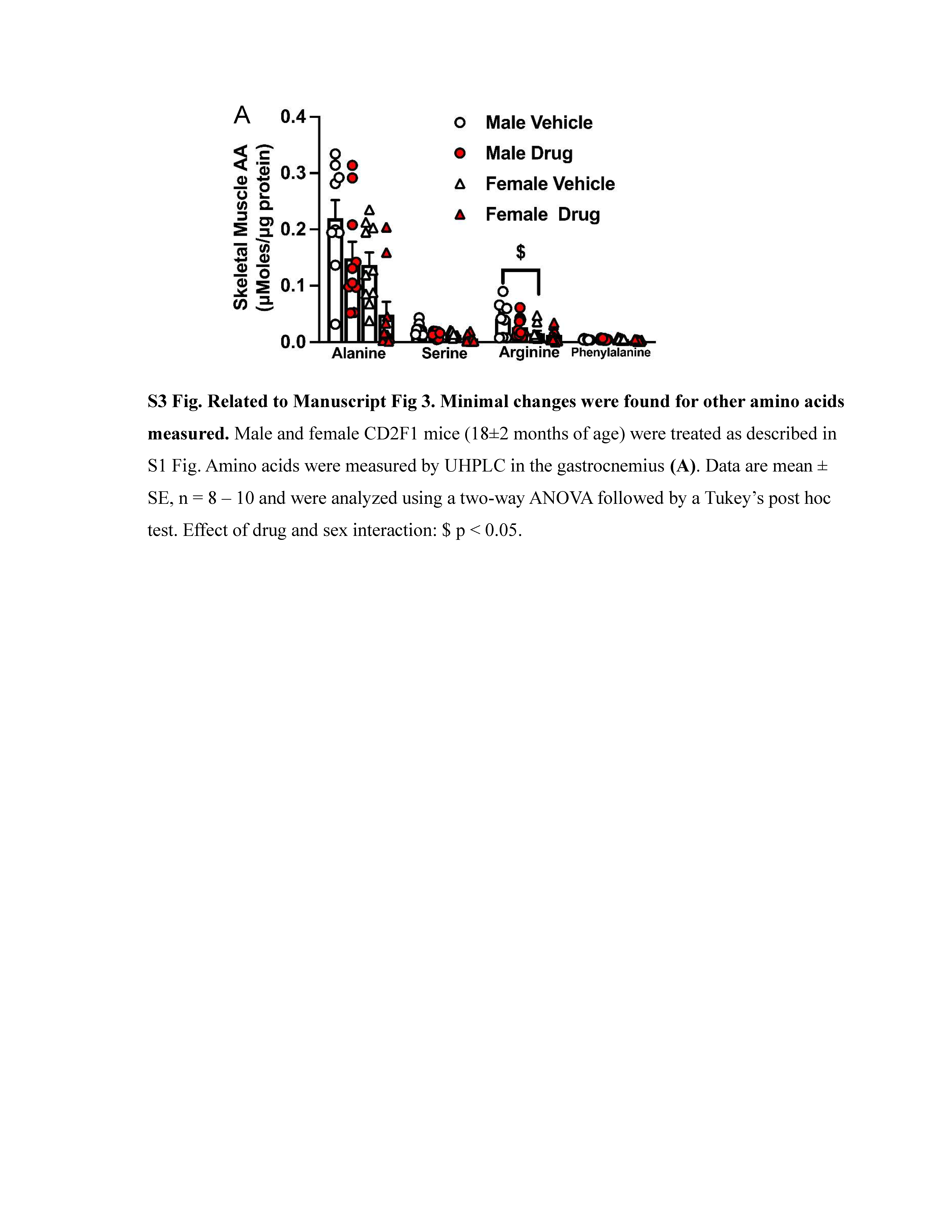

Supplement: S3 Fig — There are minimal treatment effects on alanine, serine, arginine and phenylalanine. Male and female CD2F1 mice (18 ± 2 months old) were treated as described in S1 Fig. Gastrocnemius muscle intracellular amino acid levels were measured by UHPLC. Data are mean ± SE, n = 8–10 and were analyzed using a two-way ANOVA followed by a Tukey’s post hoc test. Interaction effects of chemotherapy and sex: $ p < 0.05. (TIF) [file pone.0340647.s003.tif]

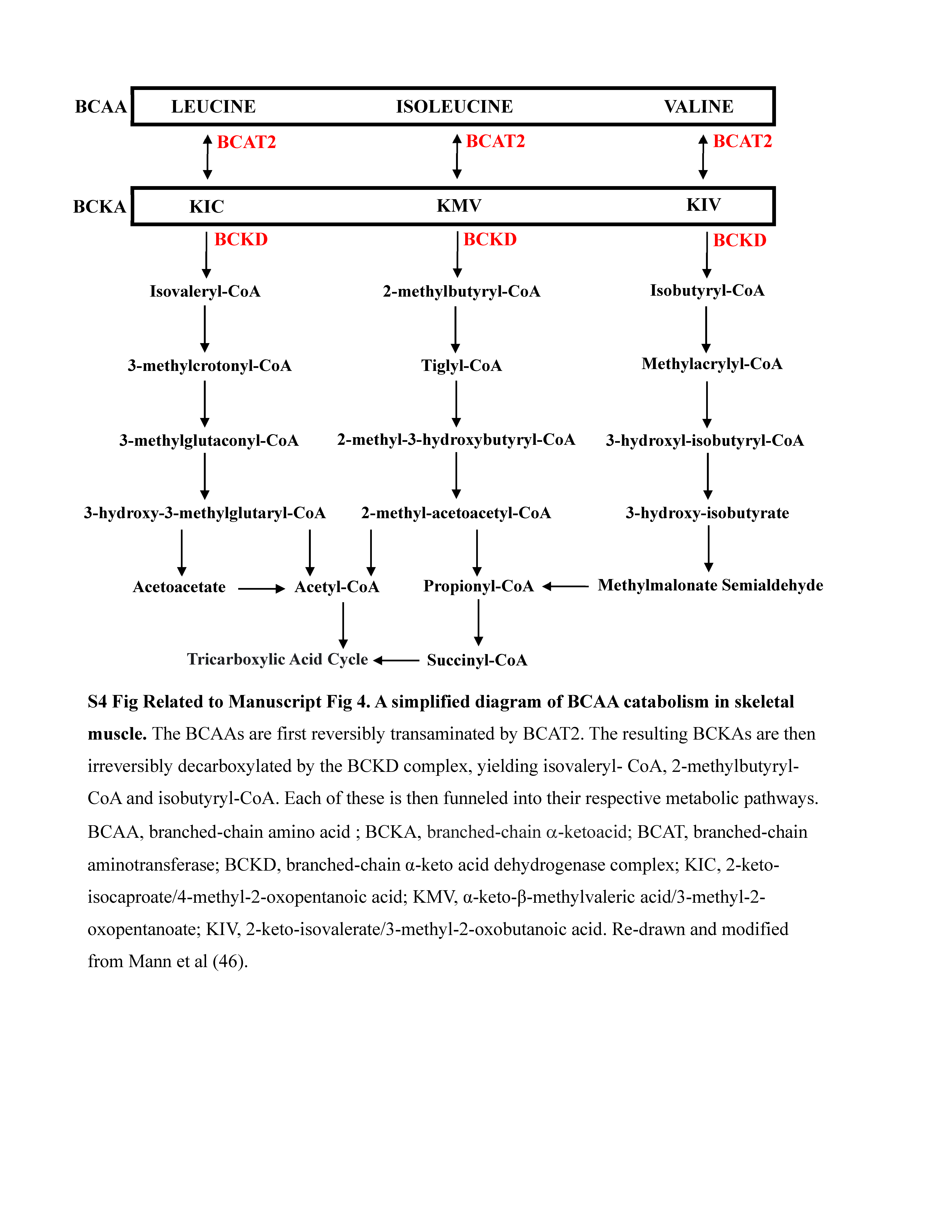

Supplement: S4 Fig — A simplified diagram of BCAA catabolism in skeletal muscle. BCAAs are first reversibly transaminated by BCAT2. The resulting BCKAs are then irreversibly decarboxylated by the BCKD complex, yielding isovaleryl- CoA, 2-methylbutyryl-CoA and isobutyryl-CoA. Each of these is then funneled into their respective metabolic pathways. BCAA, branched-chain amino acid; BCKA, branched-chain α-ketoacid; BCAT, branched-chain aminotransferase; BCKD, branched-chain α-keto acid dehydrogenase complex; KIC, 2-keto-isocaproate/4-methyl-2-oxopentanoic acid; KMV, α-keto-β-methylvaleric acid/3-methyl-2-oxopentanoate; KIV, 2-keto-isovalerate/3-methyl-2-oxobutanoic acid. Re-drawn and modified from Mann et al [31]. (TIF) [file pone.0340647.s004.tif]
