# Supplementary material for: Sex differences in cachexia outcomes and branched-chain amino acid metabolism following chemotherapy in aged mice
Source: PLoS One. 2026 Jan 12;21(1):e0340647. doi: 10.1371/journal.pone.0340647 (PMC12795360; doi:10.1371/journal.pone.0340647)
Supplement: S1 Table — (PDF) [file pone.0340647.s005.pdf]

S1 Table: Publications on Animal Studies Investigating Cancer- and Chemotherapy-Induced Cachexia

Sex / Animal	Age (Weeks)	Cancer	Chemotherapy	Body Weight Loss (~%)	Muscle Weight Loss (~%)	Ref
Male Wistar Rats	3	Walker 256	--	10	--	(1)
Male Wistar Rats	5	AH-130 Hepatoma	--	--	GA: 13<br>EDL: 18<br>TA: 12	(2)
Female Nude Mice	5	SEKI Human Melanoma	--	20	--	(3)
C57BL/6J Mice (no sex given)	5-6	Lewis Lung Carcinoma	--	25	GA: 30	(4)
Male CD2F1 Mice	6	C26 Adenocarcinoma	--	Significant body weight change	Significantly smaller gain in muscle mass	(5)
Male Balb/c Mice	6	C26 Adenocarcinoma	--	10	--	(6)
Male Balb/c and C57BL/6J Mice	6	C26 Adenocarcinoma	--	20	GA: 20<br>TA: 22	(7)
Male and Female Balb/c Mice	6	C26 Adenocarcinoma	5FU (50mg/kg) and oxaliplatin (6mg/kg), 3 injections	Cancer: 15<br>Cancer + Chemo: 22	Cancer GA: 25<br>Cancer + Chemo GA: 40	(8)
Male C57BL/6J Mice	5-7	Lewis Lung Carcinoma	--	25% less weight gained compared to control	--	(9)
Male CD2F2 Mice	6-7	C26 Adenocarcinoma	--	10	TA: 20	(10)
CD2F1 Mice (no sex given)	6-8	C26 Adenocarcinoma	--	30	TA: 40<br>GA: 40<br>QU: 50	(11)
Male C57BL/6J Mice	7	Lewis Lung Carcinoma	--	Significant Decrease	TA: ~30<br>EDL: ~40	(12)
Male CD2F1 Mice	7-8	C26 Adenocarcinoma	Cisplatin (5mg/kg), 4 injections	Cancer: 5<br>Chemo: 15<br>Cancer + Chemo: 10	Cancer GA: 15<br>Chemo GA: 15<br>Cancer + Chemo GA: 15	(13)
Male CD2F1 Mice	8	C26 Adenocarcinoma	5FU (50mg/kg), LEU (90mg/kg), CPT-11 (24mg/kg) for 5 weeks	Cancer: 30<br>Chemo: 15<br>Cancer + Chemo: 30	Cancer: GA: 25<br>Cancer: TA: 20<br>Cancer: QU: 20<br>Chemo: GA: 15<br>Chemo: TA: 10<br>Chemo: QU: 15<br>Cancer + Chemo: GA: 40<br>Cancer + Chemo: TA: 25<br>Cancer + Chemo: QU: 40	(14)
Male CD2F1 Mice	8	--	5FU (30mg/kg), LEU (90mg/kg), CPT-11 (24mg/kg) for 5 weeks	10	GA: 10<br>TA: 15<br>QU: 25	(15)
Male CD2F1 Mice	8	C26 Adenocarcinoma	5FU (30mg/kg), LEU (90mg/kg), CPT-11 (24mg/kg) for 5 weeks	Cancer: 13<br>Chemo: 15	Cancer GA: 23<br>Cancer QU: 25<br>Chemo GA: 11<br>Chemo QU: 20	(16)

Male CD2F1 Mice	8	--	5FU (30mg/kg), LEU (90mg/kg), CPT-11 (24mg/kg) for 5 weeks	--	GA: 10	(17)
C57BL/6J Mice (no sex given)	8	--	Cisplatin (2.5mg/kg), 9 injections	--	GA: 10	(17)
Female Sprague-Dawley Rats	8	--	Doxorubicin (4mg/kg), 3 injections	15	SOL CSA: 25<br>EDL CSA: 35	(18)
Male C57BL/6J Mice	8	Lewis Lung Carcinoma	--	--	TA: 30<br>EDL: 30	(19)
Male C57BL/6J Mice	8-9	--	Cisplatin (3m/kg), 4 injections	15	QU: 18<br>HL: 20	(20)
Male C57BL/6J Mice	6-10	--	Doxorubicin (15mg/kg), 1 injection	--	All Muscles: 10	(21)
Male Balb/c Mice	8-10	--	Doxorubicin (2.5mg/kg) for 4 weeks	10	TA: 10<br>EDL: 13<br>SOL: 8	(22)
Male Wistar Rats	8-12	Walker 256	--	10	--	(23)
Male Wistar Rats	8-12	Walker 256	--	2	--	(24)
Male C57BL/6J Mice	9-10	--	Doxorubicin (4mg/kg), 4 injections	10	GA: 15<br>TA: 8<br>SOL: 5	(25)
Male Sprague-Dawley Rats	10	--	Doxorubicin (15mg/kg), 1 injection	2	EDL: 2	(26)
Male C57BL/6J Mice	10	Lewis Lung Carcinoma	--	10	GA: 10<br>SOL: 15	(27)
Male Wistar Rats	10	Walker 256	--	Significant Decrease	GA: 25	(28)
Male Rats: F344/NTacfBR	10-12	Methylcholanthrene Sarcoma	--	10	--	(29)
Male and Female C57BL/6J	12	Methylcholanthrene Sarcoma	--	20	--	(30)
Male C57BL/6J Mice	12	Lewis Lung Carcinoma	--	20	QU: ~25<br>GA: ~25<br>TA: ~50	(31)
Female Wistar Rats	12	AH-130 Hepatoma	--	5	HL: 40	(32)
Male Wistar Rats	14	--	Doxorubicin (15mg/kg), 1 injection	Significant change (-30.05) from Control	EDL: 10	(33)
Male and Female C57BL/6J Mice	16	--	5FU (30mg/kg), LEU (90mg/kg), CPT-11 (24mg/kg) for 9 weeks	12	Lean Mass: 15	(34)
Male C57BL/6J Mice	18-20	Lewis Lung Carcinoma	--	5	GA: 15<br>TA: 15	(35)
Male C57BL/6J Mice	20-28	Lewis Lung Carcinoma	--	20	--	(36)
Male and Female C57BL/6J Mice	8 (Yo) and 72 (Old)	--	Cisplatin (2.5mg/kg), 9 injections	Yo Male: 13<br>Yo Female: 12<br>Old Male: 34<br>Old Female: 27	Yo Male GA: 16<br>Yo Female GA:11<br>Old Male GA: 22<br>Old Female GA: 27	(37)
Male Balb/c Mice	8-16 (Yo) and 60-80 (Old)	C26 Adenocarcinoma	--	Yo Male: 15<br>Old Male: 13	Yo Male GA: 10<br>Old Male GA: 10	(38)

Male Lister Hooded Rats	--	--	Cisplatin (4mL/kg)	6	EDL: 8	(39)
Male Wistar Rats	--	AH-130 Hepatoma	--	--	GA: 10<br>SOL: 15	(40)
Male C57BL/6J Mice	--	Lewis Lung Carcinoma	--	5	--	(41)
Male Wistar Rats and Male C57BL/6J Mice	--	AH-130 Hepatoma and C26 Adenocarcinoma	--	Rat: 20<br>Mice: 15	Rat GA: 20<br>Mice GA: 20	(42)
Male Wistar Rats	--	C26 Adenocarcinoma	--	20	GA: 20<br>TA: 20	(43)
Male Wistar Rats	--	AH-130 Hepatoma	--	25	GA: 25	(44)
Male Wistar Rats	--	AH-130 Hepatoma	--	30	GA: 25	(45)

GA, Gastrocnemius; TA, Tibialis Anterior; QU, Quadriceps; SOL, Soleus; EDL, Extensor Digitorum Longus; HL, Hindlimb; 5FU, 5-fluorouracil; CPT-11, Irinotecan Hydrochloride; LEU, Leucovorin; CSA, Cross Sectional Area; Yo, young; --, not measured/information not provided; Chemo, Chemotherapy.

REFERENCES

1. **Pizato N**, **Bonatto S**, **Yamazaki RK**, **Aikawa J**, **Nogata C**, **Mund RC**, **Nunes EA**, **Piconcelli M**, **Naliwaiko K**, **Curi R**, **Calder PC**, **Fernandes LC**. Ratio of n6 to n-3 fatty acids in the diet affects tumor growth and cachexia in Walker 256 tumor-bearing rats. *Nutr Cancer* 53: 194–201, 2005.

2. **Busquets S**, **Toledo M**, **Marmonti E**, **Orpí M**, **Capdevila E**, **Betancourt A**, **López-Soriano FJ**, **Argilés JM**. Formoterol treatment downregulates the myostatin system in skeletal muscle of cachectic tumour-bearing rats. *Oncol Lett* 3: 185–189, 2012. doi: 10.3892/ol.2011.442.

3. **Hanada T**, **Toshinai K**, **Kajimura N**, **Nara-Ashizawa N**, **Tsukada T**, **Hayashi Y**, **Osuye K**, **Kangawa K**, **Matsukura S**, **Nakazato M**. Anti-cachectic effect of ghrelin in nude mice bearing human melanoma cells. *Biochem Biophys Res Commun* 301: 275–279, 2003. doi: 10.1016/S0006-291X(02)03028-0.

4. **Liu H**, **Li L**, **Zou J**, **Zhou T**, **Wang B**, **Sun H**, **Yu S**. Coix seed oil ameliorates cancer cachexia by counteracting muscle loss and fat lipolysis. *BMC Complement Altern Med* 19: 267, 2019.

5. **Joppa MA**, **Gogas KR**, **Foster AC**, **Markison S**. Central infusion of the melanocortin receptor antagonist agouti-related peptide (AgRP(83-132)) prevents cachexia-related symptoms induced by radiation and colon-26 tumors in mice. .

6. **Weyermann P**, **Dallmann R**, **Magyar J**, **Anklin C**, **Hufschmid M**, **Dubach-Powell J**, **Courdier-Fruh I**, **Henneböhle M**, **Nordhoff S**, **Mondadori C**. Orally available selective melanocortin-4 receptor antagonists stimulate food intake and reduce cancer-induced cachexia in mice. *PLoS One* 4: e4774, 2009. doi: 10.1371/journal.pone.0004774.

7. **Pin F**, **Busquets S**, **Toledo M**, **Camperi A**, **Lopez-Soriano FJ**, **Costelli P**, **Argilés JM**, **Penna F**. Combination of exercise training and erythropoietin prevents cancer-induced muscle alterations. *Oncotarget* 6: 43202–43215, 2015. doi: 10.18632/oncotarget.6439.

8. **Ballarò R**, **Beltrà M**, **De Lucia S**, **Pin F**, **Ranjbar K**, **Hulmi JJ**, **Costelli P**, **Penna F**. Moderate exercise in mice improves cancer plus chemotherapy-induced muscle wasting and mitochondrial alterations. *FASEB Journal* 33: 5482–5494, 2019.

9. **Markison S**, **Foster AC**, **Chen C**, **Brookhart GB**, **Hesse A**, **Hoare SRJ**, **Fleck BA**, **Brown BT**, **Marks DL**. The regulation of feeding and metabolic rate and the prevention of murine cancer cachexia with a small-molecule melanocortin-4 receptor antagonist. *Endocrinology* 146: 2766–2773, 2005. doi: 10.1210/en.2005-0142.

10. **van Norren K**, **Kegler D**, **Argilés JM**, **Luiking Y**, **Gorselink M**, **Laviano A**, **Arts K**, **Faber J**, **Jansen H**, **van der Beek EM**, **van Helvoort A**. Dietary supplementation with a specific combination of high protein, leucine, and fish oil improves muscle function and daily activity in tumour-bearing cachectic mice. *Br J Cancer* 100: 713–22, 2009.

11. **Inaba S**, **Hinohara A**, **Tachibana M**, **Tsujikawa K**, **Fukada S**. Muscle regeneration is disrupted by cancer cachexia without loss of muscle stem cell potential. *PLoS One* 13: e0205467, 2018. doi: 10.1371/journal.pone.0205467.

12. **Zhang G**, **Liu Z**, **Ding H**, **Miao H**, **Garcia JM**, **Li YP**. Toll-like receptor 4 mediates Lewis lung carcinoma-induced muscle wasting via coordinate activation of protein degradation pathways. *Sci Rep* 7: 2273, 2017. doi: 10.1038/s41598-017-02347-2.

13. **Damrauer JS**, **Stadler ME**, **Acharyya S**, **Baldwin AS**, **Couch ME**, **Guttridge DC**. Chemotherapy-induced muscle wasting: association with NF-κB and cancer cachexia. *Eur J Transl Myol* 18: 139–148, 2018. doi: 10.4081/ejtm.2018.7590.

14. **Pin F**, **Barreto R**, **Couch ME**, **Bonetto A**, **O'Connell TM**. Cachexia induced by cancer and chemotherapy yield distinct perturbations to energy metabolism. *J Cachexia Sarcopenia Muscle* 10: 140–154, 2019. doi: 10.1002/jcsm.12360.

15. **Barreto R**, **Waning DL**, **Gao H**, **Liu Y**, **Zimmers TA**, **Bonetto A**. Chemotherapy-related cachexia is associated with mitochondrial depletion and the activation of ERK1/2 and p38 MAPKs. *Oncotarget* 7: 43442–43460, 2016.

16. **Barreto R**, **Mandili G**, **Witzmann FA**, **Novelli F**, **Zimmers TA**, **Bonetto A**. Cancer and chemotherapy contribute to muscle loss by activating common signaling pathways. *Front Physiol* 7: 472, 2016. doi: 10.3389/fphys.2016.00472.

17. **Huot JR**, **Pin F**, **Bonetto A**. Muscle weakness caused by cancer and chemotherapy is associated with loss of motor unit connectivity. *Am J Cancer Res* 11: 2990–3001, 2021.

18. **D'Lugos AC**, **Fry CS**, **Ormsby JC**, **Sweeney KR**, **Brightwell CR**, **Hale TM**, **Gonzales RJ**, **Angadi SS**, **Carroll CC**, **Dickinson JM**. Chronic doxorubicin administration impacts satellite cell and capillary abundance in a muscle-specific manner. *Physiol Rep* 7: e14052, 2019. doi: 10.14814/phy2.14052.

19. **Sin TK**, **Zhu JZ**, **Zhang G**, **Li YP**. P300 Mediates Muscle Wasting in Lewis Lung Carcinoma. *Cancer Res* 79: 1331–1342, 2019. doi: 10.1158/0008-5472.CAN-18-1653.

20. **Sakai H**, **Sagara A**, **Arakawa K**, **Sugiyama R**, **Hirosaki A**, **Takase K**, **Jo A**, **Sato K**, **Chiba Y**, **Yamazaki M**, **Matoba M**, **Narita M**. Mechanisms of cisplatin-induced muscle atrophy. *Toxicol Appl Pharmacol* 278: 190–199, 2014. doi: 10.1016/j.taap.2014.05.001.

21. **Hulmi JJ**, **Nissinen TA**, **Räsänen M**, **Degerman J**, **Lautaoja JH**, **Hemanthakumar KA**, **Backman JT**, **Ritvos O**, **Silvennoinen M**, **Kivelä R**. Prevention of chemotherapy-induced cachexia by ACVR2B ligand blocking has different effects on heart and skeletal muscle. *J Cachexia Sarcopenia Muscle* 9: 417–432, 2018.

22. **Lima Junior EA de**, **Teixeira AA de S**, **Silveira LS**, **Jové Q**, **Ladrón NÁ**, **Pereira MG**, **López-Soriano FJ**, **Argilés JM**, **Brum PC**, **Busquets S**, **Neto JCR**. Formoterol reduces muscle wasting in mice undergoing doxorubicin chemotherapy. *Front Oncol* 13, 2024.

23. **Lima C**, **Alves LE**, **Iagher F**, **Machado AF**, **Bonatto SJ**, **Kuczera D**, **Souza CF**, **Pequito DC**, **Muritiba AL**, **Nunes EA**, **Fernandes LC**. Anaerobic exercise reduces tumor growth, cancer cachexia and increases macrophage and lymphocyte response in Walker 256 tumor-bearing rats. *Eur J Appl Physiol* 104: 957–64, 2008. doi: 10.1007/s00421-008-0849-9.

24. **Nunes EA**, **Kuczera D**, **Brito GAP**, **Bonatto SJR**, **Yamazaki RK**, **Tanhoffer RA**, **Mund RC**, **Kryczyk M**, **Fernandes LC**. β-Hydroxy-β-methylbutyrate supplementation reduces tumor

growth and tumor cell proliferation ex vivo and prevents cachexia in Walker 256 tumor-bearing rats by modifying nuclear factor-κB expression. *Nutrition Research* 28: 487–493, 2008.

25. **Nissinen TA**, **Degerman J**, **Räsänen M**, **Poikonen AR**, **Koskinen S**, **Mervaala E**, **Pasternack A**, **Ritvos O**, **Kivelä R**, **Hulmi JJ**. Systemic blockade of ACVR2B ligands prevents chemotherapy-induced muscle wasting by restoring muscle protein synthesis without affecting oxidative capacity or atrogenes. *Sci Rep* 6: 32695, 2016. doi: 10.1038/srep32695.

26. **Quinn CJ**, **Hydock DS**. Effects of endurance exercise and doxorubicin on skeletal muscle myogenic regulatory factor expression. *Muscles Ligaments Tendons J* 7: 418–425, 2017. doi: 10.11138/mltj/2017.7.3.418.

27. **Iwata Y**, **Suzuki N**, **Ohtake H**, **Kamauchi S**, **Hashimoto N**, **Kiyono T**, **Wakabayashi S**. Cancer cachexia causes skeletal muscle damage via transient receptor potential vanilloid 2-independent mechanisms, unlike muscular dystrophy. *J Cachexia Sarcopenia Muscle* 7, 2016. doi: 10.1002/jcsm.12067.

28. **Alves CRR**, **Neves W das**, **de Almeida NR**, **Eichelberger EJ**, **Jannig PR**, **Voltarelli VA**, **Tobias GC**, **Bechara LRG**, **de Paula Faria D**, **Alves MJN**, **Hagen L**, **Sharma A**, **Slupphaug G**, **Moreira JBN**, **Wisloff U**, **Hirshman MF**, **Negrão CE**, **de Castro G**, **Chammas R**, **Swoboda KJ**, **Ruas JL**, **Goodyear LJ**, **Brum PC**. Exercise training reverses cancer-induced oxidative stress and decrease in muscle COPS2/TRIP15/ALIEN. *Mol Metab* 39, 2020. doi: 10.1016/j.molmet.2020.101012.

29. **DeBoer MD**, **Xin XZ**, **Levasseur P**, **Meguid MM**, **Suzuki S**, **Inui A**, **Taylor JE**, **Halem HA**, **Dong JZ**, **Datta R**, **Culler MD**, **Marks DL**. Ghrelin treatment causes increased food intake and retention of lean body mass in a rat model of cancer cachexia. *Endocrinology* 148: 3004–3012, 2007. doi: 10.1210/en.2007-0016.

30. **Edén E**, **Lindmark L**, **Karlberg I**, **Lundholm K**. Role of whole-body lipids and nitrogen as limiting factors for survival in tumor-bearing mice with anorexia and cachexia. *Cancer Res* 43: 3707–3711, 1983.

31. **Chen JA**, **Splenser A**, **Guillory B**, **Luo J**, **Mendiratta M**, **Belinova B**, **Halder T**, **Zhang G**, **Li YP**, **Garcia JM**. Ghrelin prevents tumour- and cisplatin-induced muscle wasting: characterization of multiple mechanisms involved. *J Cachexia Sarcopenia Muscle* 6: 132–143, 2015. doi: 10.1002/jcsm.12023.

32. **López-Soriano J**, **Argilés JM**, **López-Soriano FJ**. Lipid metabolism in rats bearing the Yoshida AH-130 ascites hepatoma. *Mol Cell Biochem* 165: 17–23, 1996. doi: 10.1007/bf00229741.

33. **de Lima Junior EA**, **Yamashita AS**, **Pimentel GD**, **De Sousa LGO**, **Santos RVT**, **Gonçalves CL**, **Streck EL**, **de Lira FS**, **Rosa Neto JC**. Doxorubicin caused severe hyperglycaemia and insulin resistance, mediated by inhibition in AMPk signalling in skeletal muscle. *J Cachexia Sarcopenia Muscle* 7: 615–625, 2016. doi: 10.1002/jcsm.12104.

34. **Englund DA**, **Jolliffe AM**, **Hanson GJ**, **Aversa Z**, **Zhang X**, **Jiang X**, **White TA**, **Zhang L**, **Monroe DG**, **Robbins PD**, **Niedernhofer LJ**, **Kamenecka TM**, **Khosla S**, **LeBrasseur NK**.

Senotherapeutic drug treatment ameliorates chemotherapy-induced cachexia. *JCI Insight* 9, 2024.

35. **Brown JL**, **Rosa-Caldwell ME**, **Lee DE**, **Blackwell TA**, **Brown LA**, **Perry RA**, **Haynie WS**, **Hardee JP**, **Carson JA**, **Wiggs MP**, **Washington TA**, **Greene NP**. Mitochondrial degeneration precedes the development of muscle atrophy in progression of cancer cachexia in tumour-bearing mice. *J Cachexia Sarcopenia Muscle* 8: 926–938, 2017.

36. **Liu H**, **Luo J**, **Guillory B**, **Chen J**, **Zang P**, **Yoeli JK**, **Hernandez Y**, **Lee I (In-gi)**, **Anderson B**, **Storie M**, **Tewnion A**, **Garcia JM**. Ghrelin ameliorates tumor-induced adipose tissue atrophy and inflammation via Ghrelin receptor-dependent and -independent pathways. *Oncotarget* 11: 32863302, 2020. doi: 10.18632/oncotarget.27705.

37. **Huot JR**, **Pin F**, **Chatterjee R**, **Bonetto A**. PGC1α overexpression preserves muscle mass and function in cisplatin-induced cachexia. *J Cachexia Sarcopenia Muscle* 13: 2480–2491, 2022. doi: 10.1002/jcsm.13035.

38. **Geppert J**, **Walth AA**, **Terrón Expósito R**, **Kaltenecker D**, **Morigny P**, **Machado J**, **Becker M**, **Simoes E**, **Lima JDCC**, **Daniel C**, **Berriel Diaz M**, **Herzig S**, **Seelaender M**, **Rohm M**. Aging Aggravates Cachexia in Tumor-Bearing Mice. *Cancers (Basel)* 14, 2021. doi: 10.3390/cancers14010090.

39. **Brierley DI**, **Harman JR**, **Giallourou N**, **Leishman E**, **Roashan AE**, **Mellows BAD**, **Bradshaw HB**, **Swann JR**, **Patel K**, **Whalley BJ**, **Williams CM**. Chemotherapy-induced cachexia dysregulates hypothalamic and systemic lipoamines and is attenuated by cannabigerol. *J Cachexia Sarcopenia Muscle* 10: 844–859, 2019. doi: 10.1002/jcsm.12426.

40. **Muscaritoli M**, **Costelli P**, **Bossola M**, **Grieco G**, **Bonelli G**, **Bellantone R**, **Doglietto GB**, **Rossi-Fanelli F**, **Baccino FM**. Effects of simvastatin administration in an experimental model of cancer cachexia. *Nutrition* 19: 936–939, 2003. doi: 10.1016/j.nut.2003.08.004.

41. **Nicholson JR**, **Kohler G**, **Schaerer F**, **Senn C**, **Weyermann P**, **Hofbauer KG**. Peripheral administration of a melanocortin 4-receptor inverse agonist prevents loss of lean body mass in tumor-bearing mice. *Journal of Pharmacology and Experimental Therapeutics* 317: 771–777, 2006. doi: 10.1124/jpet.105.097725.

42. **Penna F**, **Costamagna D**, **Pin F**, **Camperi A**, **Fanzani A**, **Chiarpotto EM**, **Cavallini G**, **Bonelli G**, **Baccino FM**, **Costelli P**. Autophagic degradation contributes to muscle wasting in cancer cachexia. *American Journal of Pathology* 182: 1367–1378, 2013. doi: 10.1016/j.ajpath.2012.12.023.

43. **Penna F**, **Costamagna D**, **Fanzani A**, **Bonelli G**, **Baccino FM**, **Costelli P**. Muscle wasting and impaired Myogenesis in tumor bearing mice are prevented by ERK inhibition. *PLoS One* 5: 13604-undefined, 2010. doi: 10.1371/journal.pone.0013604.

44. **Costelli P**, **Muscaritoli M**, **Bossola M**, **Moore-Carrasco R**, **Crepaldi S**, **Grieco G**, **Autelli R**, **Bonelli G**, **Pacelli F**, **Lopez-Soriano FJ**, **Argilés JM**, **Doglietto GB**, **Baccino FM**, **Rossi Fanelli F**. Skeletal muscle wasting in tumor-bearing rats is associated with MyoD down-regulation. *Int J Oncol* 26: 1663–1668, 2005. doi: 10.3892/ijo.26.6.1663.

45. **Costelli P**, **Carbó N**, **Tessitore L**, **Bagby GJ**, **Lopez-Soriano FJ**, **Argilés JM**, **Baccino FM**. Tumor necrosis factor-α mediates changes in tissue protein turnover in a rat cancer cachexia model. *Journal of Clinical Investigation* 92: 2783–2789, 1993. doi: 10.1172/JCI116897.

46. **Mann G**, **Mora S**, **Madu G**, **Adegoke OAJ**. Branched-chain Amino Acids: Catabolism in Skeletal Muscle and Implications for Muscle and Whole-body Metabolism. *Front Physiol* 12: 2021.
