# Supplementary material for: Sex differences in cachexia outcomes and branched-chain amino acid metabolism following chemotherapy in aged mice
Source: PLoS One. 2026 Jan 12;21(1):e0340647. doi: 10.1371/journal.pone.0340647 (PMC12795360; doi:10.1371/journal.pone.0340647)
Supplement: S2 Table — (PDF) [file pone.0340647.s006.pdf]

S2 Table – Primary Antibodies

ANTIBODY	SOURCE	DILUTION	RRID	COMPANY
MyHC-1	Mouse	1:500	AB_2147781	Developmental Hybridoma (MF-20)
Troponin	Mouse	1:400	AB_2618103	Developmental Hybridoma (JLT12)
Tropomyosin	Mouse	1:400	AB_2205770	Developmental Hybridoma (CH-1)
p-FoxO3a^{ser253}	Rabbit	1:1000	AB_2106674	Cell Signalling Tech (#9466)
p-AKT^{ser473}	Rabbit	1:1000	AB_2315049	Cell Signalling Tech (#4060)
p-S6$^{ser235/236}$	Rabbit	1:1000	AB_916156	Cell Signalling Tech (#4858)
p-S6K1^{thr389}	Rabbit	1:1000	AB_2269803	Cell Signalling Tech (#9234)
SNAT1	Rabbit	1:1000	AB_2799092	Cell Signalling Tech (#36057)
p-BCKD-E1α^{ser293}	Rabbit	1:1000	AB_2799176	Cell Signalling Tech (#40368)
BCKD-E1α	Rabbit	1:1000	AB_2800155	Cell Signalling Tech (#90198)
BCAT2	Rabbit	1:1000	AB_10792411	Protein Tech (#16417-1-AP)
MuRF1	Rabbit	1:1000	AB_11232209	Protein Tech (#55456-1-AP)
BDK	Rabbit	1:1000	AB_2548929	Invitrogen (#PA5-31455)
LAT1	Rabbit	1:500	AB_2635938	Invitrogen (#PA5-50485)
γ-tubulin	Mouse	1:10000	AB_477584	Sigma Aldrich (#T6557)
Puromycin	Mouse	1:20000	AB_2566826	Sigma Aldrich (#MABE343)
Ubiquitin	Mouse	1:500	AB_628423	Santa Cruz (#SC-8017)
