# Supplementary material for: Sex differences in cachexia outcomes and branched-chain amino acid metabolism following chemotherapy in aged mice
Source: PLoS One. 2026 Jan 12;21(1):e0340647. doi: 10.1371/journal.pone.0340647 (PMC12795360; doi:10.1371/journal.pone.0340647)
Supplement: S3 Table — (PDF) [file pone.0340647.s007.pdf]

S3Table. Daily food intake

	**Cont-Male**		**Cont-Fem**		**Drug-Male**		**Drug-female**		**Statistics**		
Day	**Mean**	**SD**	**Mean**	**SD**	**Mean**	**SD**	**Mean**	**SD**	**Trt**	**Sex**	**Int**
D2	3.7	1.3	3.5	1.1	3.0	1.3	2.8	0.9			
D3	3.5	0.8	2.5	1.0	2.4	0.9	1.6	0.5			
**D4**	**3.2**a	0.7	**2.1**b	0.4	2.5	1.0	1.9	0.5			0.005
D5	3.1	0.5	2.5	0.1	2.7	0.6	1.8	0.0		.04	
D6	4.0	1.2	2.8	0.4	3.4	0.9	2.3	0.7		.04	
D7	4.0	1.2	2.6	0.7	3.4	0.9	2.3	0.7		.04	
**D8**	3.5	1.7	2.7	0.6	3.0	1.2	2.5	0.7			
D9	3.5	0.8	3.4	1.2	2.9	1.0	2.2	0.2			
D10	3.3	0.8	2.8	0.8	2.8	0.8	2.4	0.6			
**D11**	3.3[a]	0.9	3.1[a]	0.3	2.7[a]	0.7	1.8[b]	0.5			.03
D12	4.0	1.2	3.2[a]	0.7	3.7	0.9	4.5[b]	0.4			.001
D13	4.2	1.2	3.6	0.3	3.8	0.7	3.5	0.9			
D14	4.1	1.2	3.6	0.3	3.9	0.7	3.5	0.9			
**D15**	3.8	0.7	3.3[a]	0.0	2.7	1.1	2.1[b]	0.5			.008
D16	4.4	2.0	4.0[a]	0.0	2.8	1.1	2.4[b]	0.0			.0001
D17	3.6	1.2	4.6	0.3	4.2	1.4	4.2	1.0			
**D18**	4.0	1.8	2.8	0.2	4.9	2.3	2.9	1.6			
D19	3.5	1.3	3.5[a]	0.7	3.8	1.0	1.8[b]	0.4			.0001
D20	3.2	0.5	3.5[a]	0.6	3.6	0.7	1.9[b]	0.4			.0001
D21	3.2	0.5	3.3[a]	0.4	3.6	0.7	1.9[b]	0.4			.0001
**D22**	3.5	0.8	4.3[a]	1.3	3.5	2.0	1.6[b]	0.2			.0003
D23	3.5	1.0	5.0[a]	1.7	3.5	1.5	2.4[b]	0.5			.0004
D24	4.5	1.4	4.6	0.8	4.1	1.5	4.0	0.8			
**D25**	3.6	0.6	3.5[a]	0.3	4.6	2.9	2.7[b]	0.7			.03
D26	3.8	1.1	3.5[a]	0.5	3.8	0.9	2.4[b]	0.2			.006
D27	4.0	1.1	3.1[a]	0.3	4.1	1.5	1.7[b]	0.9			.003
D28	4.1	1.1	3.1[a]	0.3	4.3	1.3	2.0[b]	0.5			.001
**D29**	4.0	0.9	3.6[a]	1.0	4.3	2.0	1.4[b]	0.2			.005
D30	3.9	1.0	3.9	0.8	4.6	2.9	3.4	1.0			
D31	4.0	0.7	4.4[a]	0.6	4.0	2.3	2.6[b]	0.4			.0001
**D32**	3.3	1.1	2.5	0.1	2.6	1.2	2.7	0.4			
D33	4.5	1.0	3.3[a]	0.0	4.1	1.2	2.7[b]	0.4			.04
D34	4.2	0.7	3.3[a]	0.0	4.6	1.0	2.8[b]	0.3		.04	.003
D35	3.9	0.8	3.3[b]	0.0	4.6	1.0	2.8[b]	0.2			.004

S3 Table. Related to Manuscript Fig 1 and Table 2. Male and female CD2F1 mice (18±2 months of age) were treated with either vehicle (control (Cont); 3.8% DMSO in saline) or a chemotherapy drug

cocktail (Drug; 50mg/kg 5FU, 90mg/kg Leucovorin, 24mg/kg CPT11) twice per week for 6 weeks. There was no main effect of chemotherapy on any of the study days. On some days, males ate more than females (sex effect). The interaction effects seen on some days were largely from chemotherapy-treated female mice consuming less than female control. Day 1 was the day of first chemotherapy treatment. Other treatment days are in bold, along with day 36 (not shown). P values of statistical analyses are shown in the column "Statistics." Cont Male: control (vehicle-treated) male; Cont-Fem, control female; Trt, treatment; Int, interaction effects of chemotherapy and sex; SD, standard deviation.
